# Prevalence of Isolated Atrial Amyloidosis in Young Patients Affected by Congestive Heart Failure

**DOI:** 10.1100/2012/293863

**Published:** 2012-03-12

**Authors:** Lia Millucci, Lorenzo Ghezzi, Giulia Bernardini, Daniela Braconi, Piero Tanganelli, Annalisa Santucci

**Affiliations:** ^1^Dipartimento di Biotecnologie, Università degli Studi di Siena, Via Fiorentina 1, 53100 Siena, Italy; ^2^Departimento di Patologia Umana e Oncologia, Università degli Studi di Siena, 53100 Siena, Italy

## Abstract

Atrial natriuretic peptide (ANP), whose amyloid is responsible of isolated atrial amyloidosis (IAA), is known to play an important role in the pathophysiology of congestive heart failure (CHF). We provide here the microscopic examination of atrial biopsies from 36 young (mean 40 years) CHF patients distinguished in idiopathic dilated cardiomyopathy (DC) affected and hypertrophic Cardiomyopathy (HC) affected, endorsing the presumptive association of early CHF with IAA. We utilized a multiple method, using Congo red (CR) staining, CR fluorescence (CRF), and immunohistochemistry to assess the presence of IAA in CHF. Immunostaining showed a moderate deposition of IAA in the atrium surrounding working myocardium with small intracellular deposits. Our findings suggest a monitoring of young CHF cases for the development of IAA. Our study also demonstrated how the concurrent use of immunohistochemistry, CR, and CRF may greatly enhance the detection of low-grade amyloid deposits.

## 1. Introduction

Congestive heart failure (CHF) is a leading cause of morbidity and mortality in developed countries [1]. In CHF, increased mechanical stress and the influence of circulating and myocardial neurohormones and cytokines result in a collective process known as *myocardial remodeling* [2]. This process involves two primary responses at the cellular level: (1) hypertrophy, dysfunction, and death of cardiac myocytes and (2) increased deposition and alteration of the cardiac extracellular matrix (ECM), often associated with amyloid deposition. Amyloid infiltration leads to ECM disruption resulting in diastolic dysfunction from progressive thickening and stiffening of the myocardium [3]. Until recently, the study of the cellular and molecular biology of heart failure focused almost exclusively on myocyte dysfunction, but novel findings on the mechanisms for accelerated amyloid disease progression and impaired prognosis associated with amyloid cardiac involvement [4, 5] gave a boost for the selection of pharmacological blockers of this detrimental phenomenon.

In this study, we focused on isolated atrial amyloidosis (IAA), whose incidence reaches 90% in the ninth decade [6, 7]. IAA is limited to the atrial myocardium and is characterized by the deposition of fibrillar material derived from atrial natriuretic peptide (ANP), a hormone secreted by atrial cardiomyocytes [8]. ANP plays an established integral role in circulatory hemodynamics, although no relationship has been demonstrated to link IAA and measures of cardiac performance, including ejection fraction. This suggests that conditions increasing ANP production do not necessarily promote atrial amyloidogenesis. Since the incidence of IAA in elderly hearts is high, ANP amyloidosis has been until now neglected and considered an unavoidable ageing symptom. On the other hand, atrial fibrosis, that increases in several pathologic conditions and mainly in CHF, has been demonstrated to create a substrate promoting atrial fibrillation, increasing conduction heterogeneity with multiple wavelets and focal sources being a possible mechanisms for the genesis of IAA also at an early age. In any case, amyloid deposition induces permanent structural alterations of the atria, disturbing myocyte contractility and conduction, and causes cellular toxicity by free radical accumulation, lipid peroxidation, and apoptosis [9]. Based on such correlations between peculiar CHF conditions and IAA aetiology, we provide the microscopic examination of human atrial biopsies from CHF patients affected by idiopathic dilated cardiomyopathy (DC) or hypertrophic cardiomyopathy (HC) and from people undergoing surgery for valve replacement and not affected by CHF (control).

Hypertrophic and dilated cardiomyopathies are important pathologies that increase myocardial mass, albeit with distinct patterns of remodelling reviewed in [10, 11]. Hypertrophic cardiomyopathy produces ventricular wall thickening without increases in ventricular volume, whereas both wall thickness and chamber volumes increase in dilated cardiomyopathy. Contractile parameters further discriminate between these pathologies, with systolic function preserved or improved in hypertrophic hearts but diminished in dilated cardiomyopathy.

Comparing the two groups of patients (CHF and control) we endorsed the presumptive association of CHF caused by different pathologies and early IAA.

The aim of this study was therefore to histopathologically characterize ANP amyloid infiltrations in young human atria affected by DC as well as HC and to establish a correlation between early IAA and CHF, pointing out the role of monitoring ANP amyloid for the diagnosis of CHF.

## 2. Materials and Methods

This study was approved by the local ethic committee, and informed consent for participation was obtained from all patients. The investigation is conformed with the principles outlined in the Declaration of Helsinki.

### 2.1. Tissue Samples

We examined 36 explanted hearts obtained during cardiac transplantation. All 36 patients (Table 1) had severe CHF: 21 had idiopathic dilated cardiomyopathy (DC) and 15 had hypertrophic cardiomyopathy (HC) (Table 1). DC was diagnosed based on electrocardiographic and chest roentgenographic findings of ventricular disfunction supported by echocardiographic or angiographic confirmation of global ventricular dilatation, and HC was diagnosed based on specific echocardiographic criteria [12]. We examined also 10 atrial biopsies from patients undergoing surgery for valve transplantation and not affected by CHF (control group). In addition, to fulfill diagnostic criteria, people older than 45 years were excluded from the study. Following cardiectomy, specimens were resected, fixed, and subjected to histologic preparation. Tissue samples were fixed in 10% phosphate-buffered formalin, embedded in paraffin, and sectioned at 4 *μ*m thickness, and standard histology with hematoxylin and Congo red (CR) staining was performed. We adopted a multiple quantitative method, using CR birefringence (CRB), CR fluorescence (CRF), and immunohistochemistry (IHC) to assess the morphometric pattern of IAA in CHF.

### 2.2. Amyloid Diagnosis

CR-stained sections were evaluated using Zeiss Axio Lab.A1 microscope with built-in polarization equipment, under polarized light to assess the presence of green birefringence polarization. Amyloid was diagnosed using CR staining method [13]. CR-stained paraffin sections were weakly counterstained with Mayer's acid hemalum. The characteristic green birefringence, when sited within the tissue structure, was taken as a proof of the amyloid presence [14–16].

### 2.3. Immunohistochemistry

Amyloid ANP was identified using the peroxidase-antiperoxidase (PAP) method using a 1/100 dilution of antisera against ANP peptide (Santa Cruz Biotechnology, Inc, CA, USA). Moreover, to classify the type of amyloid deposited in the heart, we performed immunohistochemical staining of tissues with appropriate commercially available antisera (Santa Cruz Biotechnology, Inc, CA, USA): antitransthyretin (TTR), antiamyloid A protein, and immunoglobulin light chains antibodies were also tested.

### 2.4. CR Fluorescence

When CRB was negative, additional paraffin sections were stained with CR and/or IHC for ANP in cases where amyloid deposition was clinically and/or pathologically suspected. CRF was evaluated under UV light (fluorescence microscope), and fluorescence was induced on CR-stained paraffin sections using a LED light as part of a Zeiss Axio Lab.A1 microscope with photographic equipment [14]. Filter set for detecting fluorescein isothiocyanate (FITC-blue) was used.

In case that CRF was positive, serial paraffin sections were stained with both CR and IHC for ANP, TTR, amyloid A protein, and immunoglobulin light chains. CR-stained sections were observed also by one of the investigators who was blinded to the original results.

### 2.5. Statistical Analysis

All values were expressed as mean ± SD. Continuous variables were compared by means of the unpaired Student's *t*-test. The *χ* ^2^ test with Yates correction and multivariance analysis method were used, where appropriate, to assess the association between the occurrence of DC, HC, and IAA. The Pearson correlation coefficient was used to determine the relationship between metric parameters. A value of *P* < 0.05 was considered to be statistically significant.

## 3. Results

### 3.1. Assessment of ANP Amyloidosis in CHF Heart Specimens

The presence and expression of atrial ANP in patients with CHF were investigated in myocardial biopsies from patients with DC (*n* = 21) and HC (*n* = 15), the average age being 40 and 41 years, respectively. In parallel, the presence of ANP amyloidosis was tested in a control group of patients not affected by CHF (average age 42 years).

The mean ANP level in all CHF patients was 88 pg/mL ± 10 pgl/mL versus 42 pg/mL ± 5 pg/mL in the control group (two tailed *t*-test, *P* < 0.05). On multivariate analysis, higher ANP levels were found to be associated significantly with CHF.

**Figure 1:**
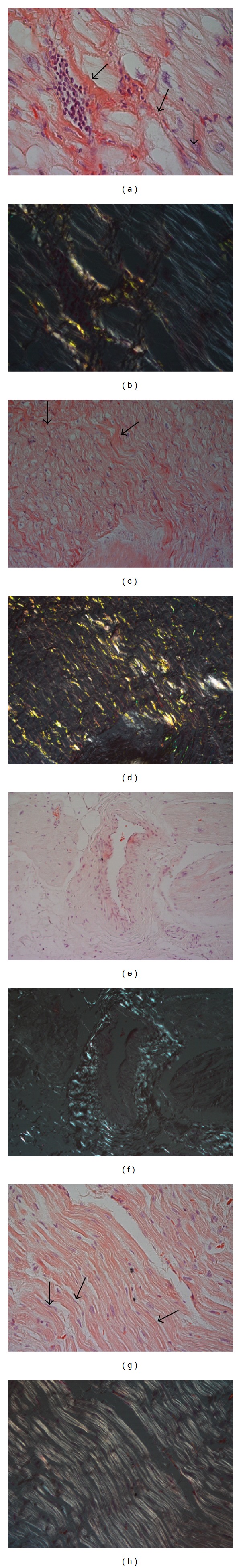
Histology of CHF hearts and CR birefringence: (a) and (b) epicardium of a DC patient with macrophage infiltrates; nonspecific abnormalities, including variations in myocyte size, myocyte vacuolation, loss of myofibrillar material, and fibrosis (arrows), can be seen. In (b) amyloid infiltration is visible as CRB. (c) Oblique and perpendicular arrangement of hypertrophied musclefibres constituting HC disarray. (d) CRB showing extensive amyloid deposition. (e) Particular of a vessel in the Purkinje of a DC patient with evident amyloid deposition. (f) CRB. (g) Disarray characterised by oblique and perpendicular arrangement of myofibril bundles in HC heart. Wide spaces among fibres are present (arrows). In (a), (c), (e) and (g) amyloid infiltration by CRB is shown. CR stain—original magnification: (a) 63x, (c)–(e) 20x, and (g) 40x.

Both DC and HC hearts demonstrated prominent histologic abnormalities, including myocyte hypertrophy, myofibrillar disarray, and fibrosis (Figure 1). None of DC or HC hearts showed dystrophic calcification but multifocal interstitial fibrosis with slight perivascular fibrosis was evidenced in HC hearts (Figure 1).

An extensive study of atrial tissues from young cardiomyopathic hearts showed that atria from dilated (DC) and hypertrophic cardiomyopathy (HC) had an evident ANP amyloid cardiac involvement.

In HC specimens, affected myofibers showed about a threefold increase in their transverse diameters and nuclear enlargement. Cells presented bizarre shapes, and the connections among cells were often in disarray (Figure 1(c)). Myocardial scarring and expansion of the collagen matrix also occurred. Scarring and disarray may constitute the substrate for arrhythmias. Other features observed were degeneration and inflammation (Figure 1(c)). Myofiber degeneration was present in the form of waviness, smudging, and vacuolation (Figure 1(g)).

In DC biopsies myocyte injury was manifested as necrosis with anucleate, hypereosinophilic myocytes with both nuclear loss and pyknosis (Figures 1(a)–1(e)). Inflammation was focal, sparse, and interstitial (Figures 1(a)–1(e)).

ANP expression was diffusely observed in HC (*n* = 11) as well as in DC (*n* = 20) patients. No amyloid deposition was observed at all in other heart districts. 2 (5.,5%) cases showed additional TTR-positive amyloid deposits. No other antibody stained amyloid. On the contrary, none of the control group specimens was positive for any kind of amyloidosis. Patients with CHF were significantly more likely to suffer from IAA (*P* < 0.05; Table 1) than those without CHF.

Table 1 summarizes the clinical characteristics of the patients and the results of statistical analysis.

#### 3.1.1. Assessment of ANP Amyloidosis in HC Heart Specimens

**Figure 2:**
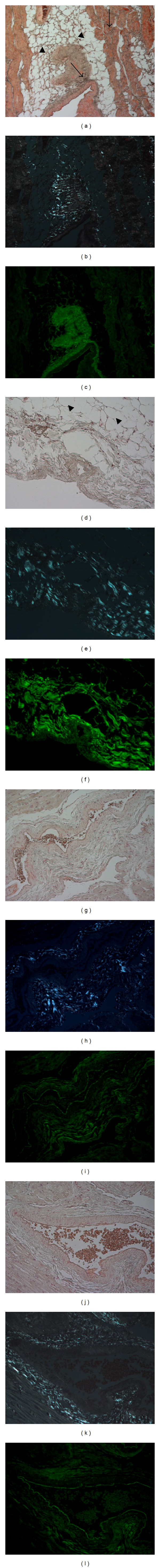
CR and anti-ANP immunostaining of HC heart specimens revealed the cooccurrence of CHF and IAA. CR-IHC-stained paraffin sections from atria of representative HC patients showing atrial amyloid under direct light ((a), (d), (g), and (j)) and polarized light ((b), (e), (h), (k)). ((c), (f), (i), (l)) CRF of ANP in the atrial myocardium. (a): Microscopic examination of a HC epicardium shows amorphous deposits that were predominantly observable in the extracellular space and interstitium (arrows) expanded by an acellular eosinophilic substance. (d)–(j) Extensive accumulation of amyloid was present between myocardial fibers and in the vessel wall of a HC patient. (a) and (d) also showed epicardial fat tissue (arrowheads). Original magnification: ((a), (d), and (g)) 20x.

In HC, ordered myofibrillar arrangement was lost in ECM invaded by amyloid deposits (Figures 1(g) and 2). Amyloid infiltration was found in the perivascular areas (Figures 2(g)–2(j)) and amyloid in HC manifested as hypertrophied, disorganized cardiac myocytes (Figures 1(c) and 1(g) and Figure 2(j)). Striking myofiber hypertrophy was present in all 15 cases. Injury was commonly interstitial, interfibrillar and in some cases perivascular. Biopsies obtained with overlying adipose tissue at the site of sampling revealed an increased penetrance of the amyloid infiltration (Figures 2(a)–2(d)). 11 amyloid deposits were immunoreactive for ANP (Figure 2) and 2 were positive for TTR. 2 HC patients were found to be not affected by amyloidosis.

Vessel involvement was found in 12 (80%) of HC samples, compared to only 2 (9.5%) of DC patients, the difference being statistically significant (*P* < 0.01).

Since patients with HC had lower amounts of amyloid in their hearts than patients with DC, in several cases CRF supported CRB to detect amyloid (Table 2).

#### 3.1.2. Assessment of ANP Amyloidosis in DC Heart Specimens

**Figure 3:**
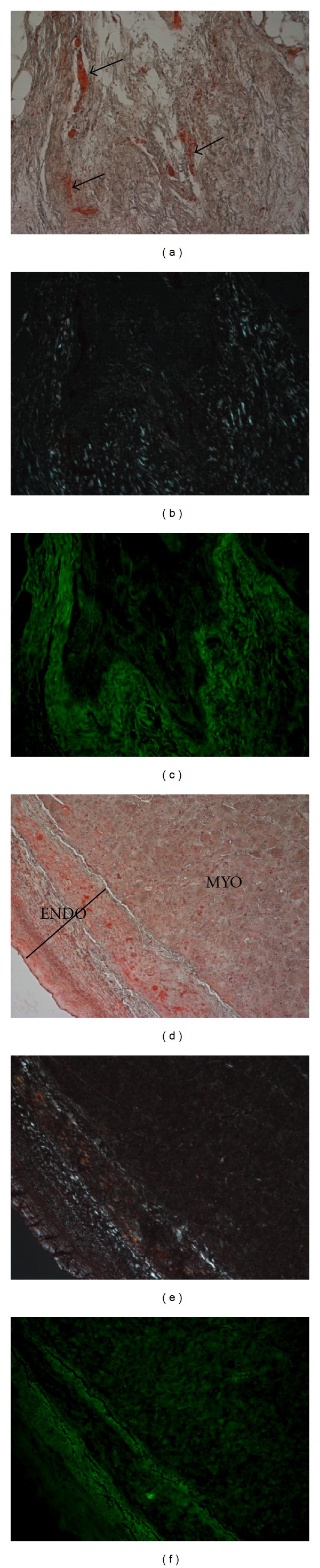
CR and anti-ANP immunostaining of DC heart specimens: CR-IHC-stained paraffin sections from atria of representative DC patients showing atrial amyloid under direct light ((a) and (d)) and polarized light ((b) and (e)). ((c) and (f)) CRF of ANP in the atrial myocardium. (a) Epicardium of a DC patient showing interstitial fibrosis (arrows), loss of nuclei, and myofiber disarray. The image also shows myofibrillar disarray and disruption of sarcomeric architecture. (d) Transmural amyloid deposition reaching through the whole myocardial layer to the endocardial border of a DC patient. Original magnification: (a) 20x, (d) 10x, and (g) 20x.

ANP expression (Figure 3) was increased in cardiomyocytes and also distributed diffusely throughout the atrium of 20 DC patients. Importantly, left atrium from patients with DC showed the presence of cardiomyopathic changes, consisting of vacuolar degeneration of myocardial fibers, often showing amyloid infiltrations indicating the involvement of amyloidosis in the cardiomyopathic process (Figures 1(a)–1(e) and Figure 3).

Microscopically, injury was present in all cases as amorphous amyloid deposition gathered around myocytes of the atrium wall (Figure 3). Transmural amyloid deposition reaching through the whole atrial layer to the internal border was visible in all 20 patients positive for amyloidosis, but small vessel disease and inflammatory cell infiltration were observed only in 2 cases. The maximum injury was transmural in all lesions (Figure 3) with endothelial necrosis.

Notably, IAA was found more commonly in women (*P* < 0.05).

Finally, patients with DC had larger amounts of amyloid in their heart specimens than patients with HC; CRF was essentially used to confirm the CRB-based diagnosis (Table 2).

## 4. Discussion

In the extensive state-of-the-art papers on CHF and left atrium functional remodelling [17–19], IAA was never mentioned. Recently, longs tanding AF has been associated with intra-atrial ANP amyloid [20], suggesting that AF-related ANP overproduction leads to localized amyloidogenesis, which in turn augments conduction heterogeneity, helping to perpetuate AF (and thereby stimulating further ANP production in a paracrine loop) [20]. In this view, atrial ANP amyloid could be considered a further negative consequence of neurohormonal disturbances. Different from what was assumed in several physiological and clinical studies, ANP plasma levels should not be considered a reliable estimation of the amount of hormone produced by the heart and even less of the overall ANP system activity because (i) plasma ANP is 1/15 to 1/20 of the total body pool [21], and (ii) plasma ANP half-life is very short (few minutes) [22]. Indeed, ANP plasma levels closely parallel the instantaneous secretion rate, and therefore likely they greatly vary in response to different pathophysiological stimuli. The discovery that ANP amyloid associated with atrial myofibrils in most human hearts with dilated or hypertrophic cardiomyopathies [23, 24] suggests IAA as a potential major pathogenic process in CHF. In the light of this, we recently demonstrated the correlation between CHF and ANP amyloidosis [25]. In the present study we presented a detailed description of deposition pattern of amyloid ANP in two groups of young CHF patients, affected respectively by DC and HC, in order to definitively establish a correlation between CHF injuries and early ANP amyloidosis. Amyloid deposits were found in 91,6% of CHF cases, 93,9% of which were also positive for ANP. At a cellular and molecular level, the focus of therapy for CHF must increasingly include the adverse changes in the ECM and cardiac amyloidosis [3]. During the past decades, medical management of heart failure has involved the blockade of the neurohormonal effects of angiotensin II, catecholamines, and aldosterone [23], but a poly pharmaceutical approach to the treatment of heart failure is emerging. However, as more antiamyloid therapies emerge, the uniform treatment of patients with multiple classes of medications is impractical and costly, so encouraging the prevention practices. The prevention of atrial ANP amyloidosis likely reduces morbidity and mortality in heart failure. The detection of specific biomarkers as natriuretic peptide plasma levels may eventually become an integral part of CHF management in the individual patient.

Atrial amyloidosis, that we have shown to be increased in CHF [25], has been demonstrated to create a substrate that promotes AF and hypertrophy with degenerative changes of myocytes is the most prominent histologic finding in all examined CHF patients. In particular, myocytolysis and ANP interstitial amyloidosis seemed to be more pronounced in the left atrial, confirming this location as an elective target for the deposition of amyloid ANP. Moreover, the inflammatory stage of the disease can involve also the entire atrial myocardium and the release of cationic proteins by the activated eosinophils may be responsible for the toxic damage of the myocardial tissue. Based on these findings, we can infer that processing of ANP precursor in the human failing heart differs from that in the normal heart, so if the protein homeostasis is altered under stress, age, or early CHF conditions, hormone aggregation may be out of control and disease-associated amyloid aggregation of hormone may occur.

Overall, our findings indicate that early IAA-related ANP deposition may occur in CHF and suggest that these latter patients should be monitored for the development of cardiac amyloidosis.

Our study also demonstrated how the concurrent use of immunohistochemistry, CRB, and CRF was superior to the CRB alone in cases in which amyloid deposits were low.

In fact, unfortunately, small amyloid deposits can be missed at light microscopy using CRB [26, 27] alone. CRF is recommended in all CRB-negative suspicious tissue samples [14]. In our work, CR staining and IHC applied together (CR-IHC) has been shown to be more sensitive than CR alone. However, a recent report suggested that CRF is the most sensitive method for the direct detection of amyloid [14]. This combined procedure increased the recognition of tissue-bound CR by virtue of its property as a fluorochrome [28], allowing an easier evaluation than CR in bright light. CR, when combined with IHC, was still visible under UV whereas CR was masked in bright light. Although not widely used, the CRF method for detecting amyloid is simple, highly specific, and sensitive; therefore, we applied the CRF technique retrospectively to paraffin sections of heart biopsies with the aim to evaluate the reported improved sensitivity compared to CR staining and to assess whether it is a suitable method if combined with IHC analysis for paraffin heart sections in order to detect also minor amyloid deposits.

In addition, unlike some previous reports where the sensitivity of CRF was reported to be low due to artifacts [29], we found complete concordance between the staining patterns of CR in bright and UV light. The high sensitivity of CRF and the specificity safe-guarded by green birefringence provide a new tool for monitoring patients at risk for amyloidosis, that is, young CHF patients as in this case. Earlier diagnosis and chemical origin identification of the amyloid will allow earlier intervention, before the development of severe organ damage [30].

## Figures and Tables

**Table 1:** Patients' characteristics: values are means ± SD or *n* (%).

Characteristic	Patients	Amyloid not present	Amyloid present	*P*	IAA	DC	HC	*P*
Total control	10	10	0		0	0	0	
Age, years, control	42.2 ± 2.2	42.2 ± 2.2	0		0	0	0	
Sex (M/F), control	4/6	4/6	0		0	0	0	
Diabetes mellitus	2	10	0		0	0	0	
Hypertension	6	10	0		0	0	0	
ANP level	42 pg/mL ± 9							
Total CHF	36	3	33		31	21	15	
Age, years, CHF	40.4 ± 4.3							
Sex (M/F)	20/16	3/0	17/16		15/16	13/8	7/8	
Diabetes mellitus	11 (31)	2 (18.2)	9 (81.8)	*P* < 0.05	1 (9)	8 (72.7)	3 (27.2)	*P* < 0.05
Hypertension	26 (73)	1 (3.8)	25 (96.2)		25 (100)	21 (84)	4 (16)	
ANP level	88 pg/mL ± 10	3	33	*P* < 0.05		21	15	*P* < 0.05
IAA	31	3	28	*P* < 0.05	31	20	11	*P* < 0.05
DC	21	1	20	*P* < 0.05	20	21	0	*P* < 0.05
HC	15	2	13	*P* < 0.05	11	0	15	*P* < 0.05

**Table 2:** Comparison between CRB, CRF, and IH methods in detecting amyloid: values are means ± SD or *n* (%).

Features	Patients	CRB negative	CRB positive	CRF negative	CRF positive
Total control	10	10 (100)	0 (0)	10 (100)	0 (0)
Age, years, control	42.2 ± 2.2	42.2 ± 2.2	0	42.2 ± 2.2	0
Diabetes mellitus					
Hypertension					
Total	36	10 (27.8)	26 (72.2)	3 (8.3)	33 (91.7)
Age, years	40.4 ± 4.3	38.5 ± 1.5	42.2 ± 8.7	27 ± 2.1	40.8 ± 3.9
Sex (M/F)	20/16	4/6	16/10	2/1	18/15
Disease					
Hypertrophic cardiomyopathy	15 (41.7)	7 (46.7)	8 (53.3)	2 (13.3)	13 (86.7)
Idiopathic dilated cardiomyopathy	21 (58.3)	3 (14.3)	18 (85.7)	1 (4.8)	20 (95.2)
Immunohistochemistry					
ANP negative	5 (13.9)	3 (60)	2 (40)	1 (20)	4 (80)
ANP positive	31 (86.1)	7 (22.6)	22 (77.4)	0 (0)	31 (100)
